# The impact of atmospheric, lunar and superstitious factors on the cause of spontaneous pneumothorax

**DOI:** 10.12669/pjms.41.11.12659

**Published:** 2025-11

**Authors:** Huseyin Fatih Sezer, Aykut Elicora, Salih Topcu

**Affiliations:** 1 Huseyin Fatih Sezer, Department of Thoracic Surgery, Kocaeli University Medical Faculty, Kocaeli, Turkey; 2 Aykut Eliçora, Department of Thoracic Surgery, Kocaeli University Medical Faculty, Kocaeli, Turkey; 3 Salih Topcu, Department of Thoracic Surgery, Kocaeli University Medical Faculty, Kocaeli, Turkey

**Keywords:** Astrological, Lunar cycle, Pneumothorax, Superstitious beliefs, Zodiac signs

## Abstract

**Objectives:**

Many causes have been put forward in the etiology of pneumothorax, but the specific atmospheric factors causing this condition have been a popular topic of discussion. Our study aimed to investigate the effects of atmospheric parameters on the occurrence of spontaneous pneumothorax, as well as the effects of the lunar cycle, important astrological events, and superstitions.

**Methodology:**

We conducted a retrospective review of 302 patients with primary spontaneous pneumothorax who applied to Kocaeli University Hospital between January 2013 to December 2022. The study time period was divided into two as days with and without pneumothorax, and the effects of climatic parameters, the moon, important astrological events, zodiac signs, and superstitions such as Friday the 13th on spontaneous pneumothorax were investigated.

**Results:**

It was determined that between days with and without pneumothorax, the maximum temperature on the day of the event, as well as one and two days before, and the minimum temperature on the day of the event, as well as one, two and three days before, were statistically significantly low. Although numerical differences in other parameters, no statistically significant difference was detected. On lunar days, the highest number of pneumothorax cases was observed on the 28th day, 17 (13.7%) and the lowest number of pneumothorax cases was observed on the 13th day, 2 (1.6%) (p=0.310). The highest number of pneumothoraces was in the waning crescent phase 68 (%25.37); the lowest number of pneumothoraces was in the first quarter phase 6 (%2.99). No significant difference was found in pneumothorax numbers in relation to the date ranges of zodiac signs or superstitions.

**Conclusions:**

Atmospheric parameters are associated with the occurrence of pneumothorax. The effect of the moon, on spontaneous pneumothorax increases during certain periods. Astrological conditions, zodiac date ranges, and superstitions are not associated with the occurrence of spontaneous pneumothorax.

## INTRODUCTION

Despite the explanation of many pathophysiology’s, there are still some superstitious, popular, astrological, and mystical beliefs related to health within societies, which are seriously accepted by a certain segment of society. Although we can partially separate the position of the moon from these situations, some beliefs include lunar and solar eclipses, Friday the 13th, and the connection between zodiac signs and health conditions. Although there are individual studies in the literature examining superstitious beliefs in surgical contexts, most of the scientific studies on the subject are related to the state of the moon. Regarding this intriguing situation, it is reported that the lunar cycle may have an effect on human life and may affect physiological and psychological parameters.^1^-^4^ Examples of situations that may be affected by the lunar cycle include psychological disorders, childbirth and miscarriage, trauma, cardiovascular disease, intracranial aneurysm, number of surgeries, emergency department visits, and kidney stones.^4^-^9^

Pneumothorax can be defined as the presence of air in the intrapleural space. Many causes have been put forward in the etiology of pneumothorax, and which atmospheric factor may cause this condition has been widely discussed in many studies.^10^ Many studies have been reported that are either in agreement or in contradiction with one another. However, no studies on superstitions were encountered in the literature review, and only two limited studies were found on the effect of the lunar cycle on spontaneous pneumothorax.^2^,^11^ In these studies, the cause of pneumothorax was explained by the gravitational force causing the formation and rupture of bullae at the apex of the lung, also by the moon indirectly influencing pneumothorax through climatic conditions.^2^,^11^ The effect of moon phases or important astrological solar and lunar events, superstitious beliefs, and dates important for astrology has not been examined.

In our study, the effects of atmospheric parameters on spontaneous pneumothorax formation and the effects of the lunar cycle, important astrological events, and superstitious beliefs were investigated. While there are many similar publications regarding the climatic parameters affecting the formation of spontaneous pneumothorax, studies examining the lunar cycle are extremely limited. Moreover, there is no study yet that reveals the contradictions between astrology, superstition, and medical literature; therefore, our study will be the first of its kind.

## METHODOLOGY

In this retrospective study involved 867 patients with primary spontaneous pneumothorax who applied to Kocaeli University hospital between January 2013 to December 2022. After exclusion criteria finally data of 302 patients were analyzed. Epidemiological parameters, daily pressure, temperature, humidity, wind, precipitation, lunar days, lunar cycle, important solar and lunar events (such as solar eclipses, lunar eclipses, and special events like super full moon and micro-new moon-blue moon-black moon), Friday the 13th, as well as zodiac dates that coincide with the event day were analyzed. The relationship between pneumothorax and atmospheric parameters was analyzed daily for three days before the day of the event and the moon phases are divided into eight phases. Pneumothorax was detected by radiological methods, and all of them were included in the study regardless of their size. Instances when at least two patients applied within a period of three consecutive days were accepted as a pneumothorax cluster. Altitudes up to 120 m above sea level were accepted as equivalent to sea level for certain measurements or purposes.

### Ethical considerations:

All procedures involving hu-man subjects were performed in accordance with the ethical standards of the institutional and national re-search committee, the 1964 Helsinki declaration, and its later amendments. This study adheres to the STROCSS criteria for reporting cohort studies in surgery. Written and verbal informed consent was obtained from all patients.

### Ethical Approval:

It was granted by the Kocaeli University Institutional Ethics Board Review (Approval No: 2024/128, Dated: 28.03.2024).

### Inclusion & Exclusion Criteria:

Patients who were living in Kocaeli and 40 km surrounding areas and with spontan pneumothorax were included. The tested positive during the COVID-19 intensive period, patients with missing data, those who did not have a first pneumothorax during the study dates, those who did not apply on the day the symptoms started, and those under the age of 18 were excluded from the study.

### Data collection and analysis:

Patient data were anonymised and accessed through the hospital information system, radiological imaging systems, and telephone patient interviews. Meteorological data were obtained from the Kocaeli Meteorology Directorate. Information on lunar cycles and phases were obtained from the website https://www.timeanddate.com/, and important lunar and solar information (for example, solar and lunar eclipses) was obtained from the Kandilli Observatory http://www.koeri.boun.edu.tr/. The dates on the website https://tr.wikipedia.org were used to determine the dates of the zodiac signs.

### Statistical analysis:

Statistical analyses were performed using IBM SPSS 29.0 (IBM Corp., Armonk, NY, USA) and MedCalc 14 (MedCalc Software, Ostend, Belgium). The normality assumption was assessed using Kolmogorov-Smirnov test. Continuous variables were expressed as the median and the interquartile range (IQR) since the normality assumption did not hold. Categorical variables were reported as counts and percentages. Comparisons between groups were performed using the Mann-Whitney U test. Associations between categorical variables were examined using the Chi-square test with Bonferroni correction. Odds ratios were computed by binary logistic regression. Receiver-operating characteristic (ROC) analysis was performed to compute the area under the curve (AUC), sensitivity, specificity, and cut-off values. A p-value of <0.05 was considered statistically significant.

## RESULTS

Of 302 patients, 248 (82.1%) were male and 54 (17.9%) were female. The median age of the patients was 24 years. There were 179 (59.3%) patients who had a smoking history. 257 participants (85.1%) had no respiratory system disease in their medical history. Pneumothorax was observed in 174 cases (57.6%) in the right hemithorax and in 128 cases (42.4%) in the left hemithorax. Pneumothoraces occurred in 268 (7.3%) days; 116 clusters were observed during this period, with a number of two to six, and the mean number of patients in clusters was 2.60±0.99.

It was observed most in winter 95(31.5%) and spring 91(30,1%) and least in summer 48(17.9%) (p=0.003-0.007), and it was also seen more in the older group in winter (p=0.001). As expected, there was a significant difference in climate parameters between seasons. (Table-I) It was observed the most in April, 40(13.2%), and the least in June, 12(4%) and there was a statistically significant difference between months (p=0.02).

**Table-I:** Pneumothorax Frequency in Seasons and Months.

Season	n^1^	n^2^	%(n^1^)	p	
Spring^1^	91	84	30,1	0,007 (1-2)	
Summer^2^	52	48	17,2		
Autumn^3^	64	57	21,2	0,003 (3-4)	
Winter^4^	95	79	31,5		
Total	302	268			
	n^1^	Total Pneumothorax Number			
	n^2^	Only Day with Pneumothorax			
Months	n	%	p(all)		
January	37	12,3	0,002		
February	28	9,3			
March	15	5			
April	40	13,2			
May	36	11,9			
June	12	4			
July	23	7,6			
August	17	5,6			
September	17	5,6			
October	20	6,6			
November	27	8,9			
December	30	9,9			
		p	0,002		
n	Number of Pneumothorax				

### Results Related to Climatic Parameters:

It was determined that between days with and without pneumothorax; maximum temperature on the day of the event and one and two days before, minimum temperature on the day of the event and one, two and three days before, humidity difference on the day of the event was statistically significantly low, although there were numerical differences in other parameters, no statistically significant difference was detected (Table-II, Fig.1).

**Table-II:** Relationship between Atmospheric Parameter Values and Pneumothorax.

Pnx	Time	Max P	Max T	Min P	Min T		Max H		Min H		W	R		P dif		H dif		T dif	
All	Same Day	1008,4	21,8	1004,55	12,4		91		48		6,7	2,204		3,4		40		9,2	
-	Same Day	1008,3	22	1004,5	12,6		91		48		6,7	2,199		3,4		40		9,1	
+	Same Day	1009,4	20,9	1005,25	10,5		91		47		6,2	2,263		3,6		43		9,45	
	p	0,07	***0,008***	0,109	***0,001***		0,431		0,214		0,149	0,96		0,387		***0,027***		0,213	
All	24 hours ago	1008,4	21,8	1004,55	12,4		91		48		6,7	2,204		3,4		40		9,2	
-	24 hours ago	1008,35	22	1004,5	12,6		91		48		6,7	2,187		3,4		40		9,1	
+	24 hours ago	1009,15	20,3	1005,15	10,75		92		47		6,3	2,422		3,7		42		9,4	
	p	0,14	***0,006***	0,57	***0,001***		0,051		0,861		0,099	0,66		0,108		0,224		0,463	
All	48 hours ago	1008,4	21,8	1004,55	12,4		91		48		6,7	2,204		3,4		40		9,2	
-	48 hours ago	1008,4	22	1004,5	12,5		91		48		6,7	2,217		3,4		40		9,2	
+	48 hours ago	1008,85	20,1	1004,7	11,45		92		47		6,2	2,043		3,8		40,5		8,95	
	p	0,43	***0,009***	0,669	***0,001***		0,093		0,864		0,328	0,503		0,053		0,885		0,92	
All	72 hours ago	1008,4	21,8	1004,55	12,4		91		48		6,7	2,204		3,4		40		9,2	
-	72 hours ago	1008,4	21,9	1004,6	12,5		91		48,5		6,7	2,223		3,4		40		9,1	
+	72 hours ago	1008,7	21,2	1004,35	11,4		91		46		6,2	1,968		3,8		40		9,5	
	p	0,92	0,055	0,438	***0,007***		0,783		0,419		0,761	0,999		0,073		0,654		0,447	
												
	P: Pressure (hPa)		H: Humidity (%)			R: Rain (kg/m^2^)				Max: Maximum					dif: Difference				
	T:Temperature (C^0^)		W: Wind (m/sn)							Min: Minimum									

**Legend for Fig.1:**
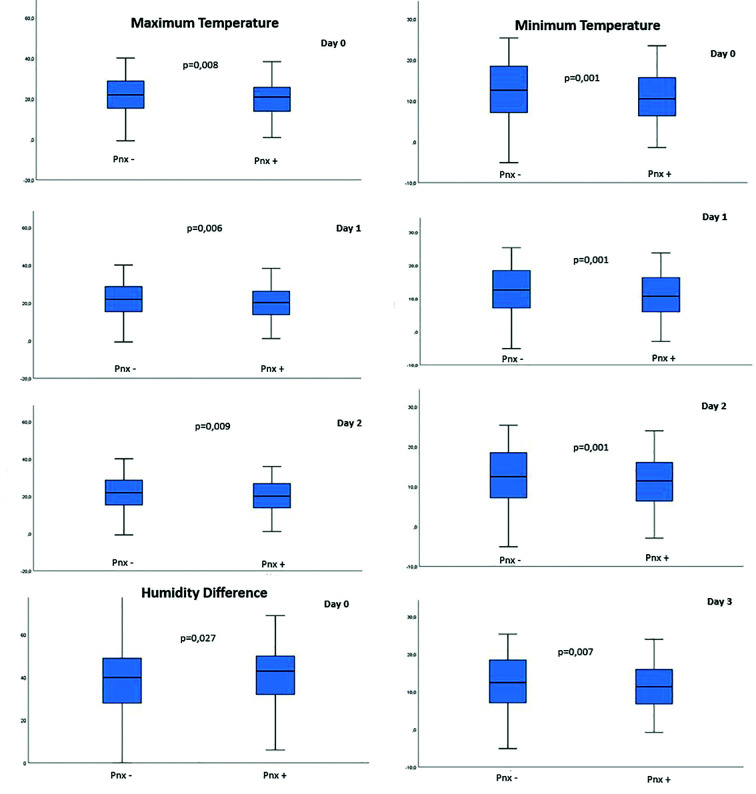
Meaningful Atmospheric Parameters (Maximum Temperature, Minimum Temperature and Humidity Difference). Day 0: Same Day. Day 1: 1 Day Ago Day2: 2 days ago Day 3: 3 days ago

It was observed that a 1°C decrease in maximum temperature increased the risk of pneumothorax by 1.94% (p=0.013), and a 1°C decrease in minimum temperature increased the risk of pneumothorax by 3.62% (p=0.001). Since the study on humidity difference was not statistically significant, numerical values were not given. A maximum temperature of 24.2°C and below, a minimum temperature of 15.6°C and below, and with a mem difference above 42% were significant in terms of pneumothorax formation.

### Results Related to the Lunar Cycle:

There was no significant difference in terms of epidemiological characteristics and climate parameters (pressure p=0.185, humidity p=0.984, wind p=0.227, temperature p=0.981, precipitation p=0.055) when comparing time periods associated with the lunar cycle to those associated with major lunar-solar events.

On lunar days, the highest number of pneumothoraxes was observed on the 28th day 17(%13.7) and the lowest number of pneumothoraxes was observed on the 13th day 2(%1.6) (p=0.310). An increase in the number of pneumothoraxes was observed between days 9-11, 20-22, 27-29. The highest number of pneumothoraces was in the waning crescent phase [68 (25.37%)], while the lowest number in the first quarter phase [6 (2.99%)]. Additionally, the order was the same in terms of the number of pneumothoraces per day (8.59% - 4.84%, respectively). No significant difference in pneumothorax frequency was found between lunar phases (p=0.885). (Table-III-Fig.2) Pneumothorax was observed on 10 (8.13%) of the full moon days. Seven (5.7%) (p=0.591) pneumothorax cases were detected one day after the full moon and three (2.4%) (p=0.052) pneumothorax cases were detected one day before the full moon.

**Table-III:** Frequency of pneumothorax in lunar day/stage.

Lunar Day	n	%	p (all)	Lunar Phase	n	%	%^*^	p
1	8	6,50%	0,31	New Moon	10	3,73	8,06	0,885
2	9	7,30%		Waxing Crescent	56	20,9	7,1	
3	10	8,10%		First Quarter	6	2,24	4,84	
4	8	6,50%		Waxing Crescent	54	20,15	6,82	
5	11	8,90%		Full Moon	10	3,73	8,13	
6	8	6,50%		Waning Moon	56	20,9	7,14	
7	6	4,80%		Last Quarter	8	2,99	6,45	
8	8	6,50%		Waning Crescent	68	25,37	8,59	
9	12	9,80%			n	Pneumothorax		
10	13	10,60%			^*^	Number per day		
11	14	11,40%						
12	8	6,50%						
13	2	1,60%						
14	6	4,90%						
15	8	6,50%						
16	6	4,90%						
17	6	4,90%						
18	10	8,10%						
19	7	5,60%						
20	11	8,90%						
21	12	9,70%						
22	10	8,10%						
23	6	4,80%						
24	8	6,50%						
25	8	6,50%						
26	6	4,80%						
27	11	8,90%						
28	17	13,70%						
29	13	10,50%						
30	6	9,10%						
n	Pneumothorax Number							

**Legend for Fig.2:**
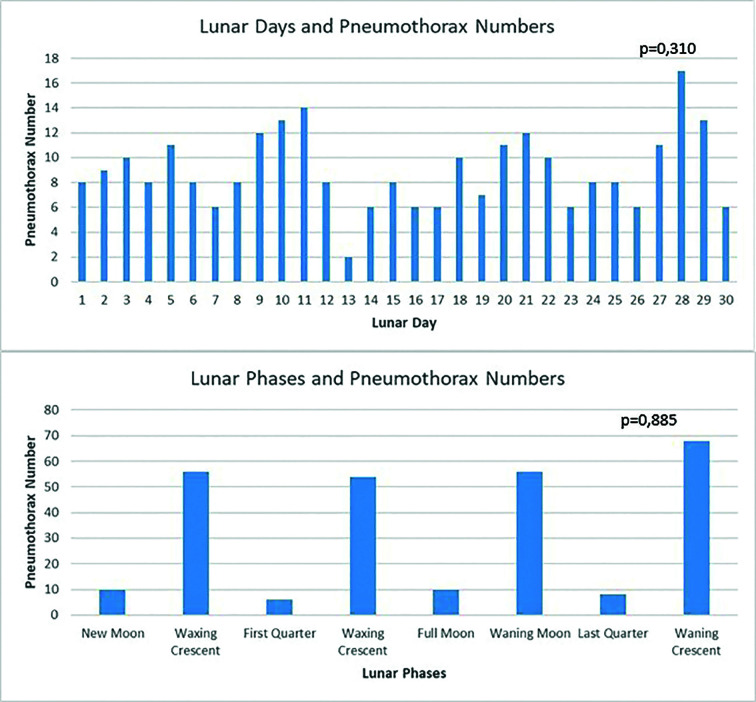
Numbers of Pneumothorax in Lunar Days and Phases.

### Astrology Related Results:

There were 89 (2.4%) significant sun-moon events, 22 (0.6%) of which were solar eclipses and 18 (0.5%) were lunar eclipses. The pneumothorax was observed on five days (5.6%) of the days with astrological events (one solar eclipse, one lunar eclipse, and three significant moon states) (p=0.671). Pneumothorax was observed one day before the event in 3 (3.4%) of the astronomical events (one solar eclipse and two lunar events) (p=0.996), but not one day after. Black moon did not occur in 2013 and 2021; there were 2 blue moons in 2018; there were three black moons in 2014; there were no blue moons in 2014 and 2017; but no significant difference was found between these years in terms of the number of days with pneumothorax.

### Superstitions and Zodiac Signs Results:

There were 17 instances of Friday the 13th; none of them had pneumothorax. There was one instance of a full moon and Friday the 13th together, and no pneumothorax was observed then either. There was one solar eclipse and micro moon together on Friday the 13th. No pneumothorax was observed. The worst-case scenario: Friday the 13th and full moon together happened once, and no pneumothorax was observed on that day. Pneumothorax was observed the most in the 33 (12.31%) date range of Taurus and Capricorn, and the least in the 11 (4.1%) date range of Virgo, no significant difference was found between the zodiac signs’ date range in terms of pneumothorax numbers (p=0.881). (Table-IV).

**Table-IV:** Pneumothorax Numbers in Zodiac Dates.

Zodiac Sign	n	%	p	Date Range
Scorpio	19	7,1	0,881	October 24 - November 22
Leo	18	6,72		July 23 - August 23
Pisces	23	8,58		February 20 - March 20
Virgo	11	4,1		August 24 - September 23
Taurus	33	12,31		April 21 - May 20
Gemini	23	8,58		May 21 - June 21
Aries	23	8,58		March 21 - April 20
Aquarius	23	8,58		January 21 - February 19
Capricorn	33	12,31		December 22 - January 20
Libra	21	7,84		September 24 - October 23
Sagittarius	22	8,21		November 23 - December 21
Cancer	19	7,1		June 22 - July 22
n	number			

## DISCUSSION

This study has revealed the relationship between climate parameters, lunar cycle, important astrological events, superstitions, zodiac signs, and pneumothorax formation in the Kocaeli region. Lunar cycle, important lunar sun events, and zodiac dates were not statistically effective, and lunar day was partially effective, while temperature and humidity were effective. Pneumothorax was significantly higher in winter and spring. While there are many similar publications in terms of climate parameters, there are two limited studies related to the lunar cycle. In addition, there is no study on astrological events, zodiac signs, and superstitions. Therefore, our study will be a reference for future researchers to compare with.

It is reported that the effects or changes of pressure, temperature, humidity, wind, or storm are differently related to either the occurrence of spontaneous pneumothorax or the periods (month and season) in which they are frequently observed (Table-V).^10^-^24^ It has been claimed that the reasons for the contradiction in the studies may be design , population , comprehensiveness of the data, localization, number of patients, altitude, and environmental factors.^12^,^13^,^21^ When we compare our own study with the studies conducted in our country to minimize these factors, we encounter different results; we think that the reason for the contradiction is the design and data coverage.

**Table-V:** Studies on Atmospheric Parameters and Their Results.

Author	Range^*^	n^**^	Parameters	Cluster	Altitude	Significant Detected Parameters	Dominant Month/Season
Özpolat et al.[13]	10	669	T, P, T-P (d),R	2,5±0,8	High	R (D0-1-2), low P (D0-1)^^^ , low T D(0)	There is no difference
Suarez et al.[10]	2	69	T, M, S,P, Ws		Sea side	P (only 7-10 mbar rise)	May/Spring - Winter
Çelik et. al. [28]	3	159	P-T-M		Sea side	No Meaningful Parameters	june-november/Autumn
Arai et al. [11]	6	131	P, T, M		High	T-P	Late of Autumn -Summer
Tülüce [22]	5	168	W, M, T, P, R	2,1±0,6	Sea side	M	November/Autumn
Bozkurt et. Al [23]	3	95	T, P, M, R, W	2,38±0,59	High	M	September-February / Autumn-Spring
Zhang et al.[14]	3	337	P, T, M, W , Local Calendar		High	low P-high T [D(0)]	April/Autumn
Chen et al. [29]	4	8575	R, T, M, P, S		Sea side	No Meaningful Parameters	There is no difference
Ozenne et al. [24]	5	165	P, T, M		Sea side	M	Summer-Winter
Ogata et al. [21]	6	110	P-T-M-W		Sea side	Ws	September/Autumn
Bulajich et. al.[16]	5	659	P,T		Sea side	Air phase	December/Autumn
Scott et al. [15]	5,5	192	P		Sea side	P	
Boulay et al.[25]	3	146		2,7	Sea side		
Kaneko et al.[17]	10	106	P,T,M,W,S		Sea side	T-M-S [D(2)]	May-July/ Summer
Smit et al. [18]	2	115	P -T		Sea side	T D(1),St [D(1)-(2)]	
Alifano et al.[12]	4	294	P,T, St	3.2 ±1.8	Sea side	St, P D(1)	There is no difference
Aissa et al.[19]	5	200	T,P,M,S,R,St		Sea side	T [(D(0)-(1)-(2)], M (D1), fırtına (D2), S[(D(0)-(1)-(2)]	Summer
Heyndricks et al.[30]	4,5	106	P,T,M,S,R,W		Sea side	No Meaningful Parameters	There is no difference
Oruç et al. [20]	5	551	W,M,T,P,R,T-P (d)		High	high T, low M [D(0)]	September/ No season difference
^*^ Year ^** Number of Patients^			T: Temperature (d): difference	S: Sunlight P: Pressure		M: Moisture St: Strom Ws:Wind speed R: Rain	^^^(- R) inversely proportional
							D(0): Same Day D(1): 1 Day Ago D(2): 2 days ago D(3): 3 days ago D(4): 4 days ago

Lung bullae are not affected by pressure changes when they are in contact with the atmosphere, but if their connections are cut off, the volume of the bullae will increase with the decrease in atmospheric pressure. This increase becomes important in terms of rupture risk as the exposure time increases.^16^ Another suggested reason for the effect of pressure is that the transpulmonary pressure gradient caused by the decrease in pressure may stretch the bullae-bleb wall and cause rupture.^12^ Suarez et al. reported that pressure increases of only 7-10 mbar (no correlation in the decrease) may be a factor.^10^ In our study, no relationship was observed between maximum pressure, minimum pressure, and pressure difference in relation to spontaneous pneumothorax. This may be due to the difference in altitude between our study and other studies, as well as our analysis of primary spontaneous pneumothorax (design difference). In this regard, Araz et al.^11^ also reported that the difference in altitude may be a factor. Similar to our study, there was no relationship between pressure and spontaneous pneumothorax in the majority of studies conducted at sea level and nearby altitudes (Table-V).

It is predicted that the air volume in the body will increase in hot and humid conditions and that bullae-bleb rupture will increase secondary to this.^17^ Kaneako et al. suggested that warm fronts will decrease atmospheric pressure, and thus implied that it may have an indirect effect on spontaneous pneumothorax.^17^ In some studies, such as Suarez et al.^10^, the limited variability of the average annual temperature in the location has been interpreted as a disadvantage in demonstrating the effect of temperature, and therefore, no significant result could be obtained.^10^ In our study, contrary to the literature, it was determined that the decrease in temperature was a significant factor influencing the results on the same day, and up to three days before.

However, it does not seem possible to explain a mechanism by which a temperature change alone increases the frequency of pneumothorax, and we could not find a clear and satisfactory answer in the literature review we conducted on the subject. This situation will be clarified with further studies on this subject. It is reported that bronchoconstriction will occur with the humidification of the air, and this may play a role in the formation of pneumothorax.^16^,^23^,^24^ The effect of wind is that it creates exposure to pollen, allergens, and air pollutants, and then causes air trapping in the bullae secondary to bronchospasm.^21^ It is thought that pneumothorax may be observed one-two days later due to sudden changes in atmospheric parameters after thunderstorms.^18^ In our study, it was determined that the difference in humidity was significant, whereas the wind was not significant. It can be thought that humidity is effective in the bronchoconstriction mechanism and may be effective in coughing. Although the storm changes some parameters while causing pneumothorax, we think that both the amount of these changes and the duration of exposure, localization, and altitude may be effective.

Aissa et al.^19^ presented numerical data not only on whether climate parameters affect the formation of pneumothorax, but also on how much they do. In the study, the risk of pneumothorax increases by 3.7% with a 1°C increase in temperature, 1.6% with a 1% decrease in humidity, and 14% with a one-hour increase in daylight hours. An average temperature above 20°C was found to be associated with a 60% higher risk of pneumothorax formation.^19^ In our study, we found that a 1°C decrease in maximum temperature increases the risk of pneumothorax by 1.94%, and a 1°C decrease in minimum temperature increases the risk of pneumothorax by 3.62%. For maximum temperature of 24.2°C and below minimum temperature of 15.6°C and below, and humidity difference above 42%, these were significant values in terms of spontaneous pneumothorax formation. The possible reasons for the difference between Aissa et al.^19^ and our study are that the climate conditions of the studied region are warmer and closer to the equator, and the temperature difference is less variable between seasons than in our region.

Some studies have analyzed atmospheric parameters in the days before pneumothorax incidence.^12^,^13^,^17^-^19^ (Table-V) There is no satisfactory explanation this issue in the literature review. Smitt et al.^18^ stated that the onset of complaints would occur a few days after the rupture of the alveolar structure, Scott et al.^15^ emphasized the importance of repeated exposures. In our study, only the temperature decrease was significant among the parameters before the day of the event. (Table-II) We think that the duration of exposure is important and that exposure may trigger some mechanisms at the cellular level. Pneumothorax may occur after the onset of exposure, and symptoms may even occur after a period of time following the formation of pneumothorax.

The days when at least two patients presented to the clinic within three consecutive days are considered a pneumothorax cluster. The clustering amounts are important for the homogeneous distribution of cases in time or for observing suspected atmospheric events. Boulany et al. reported that 60% of the cases were in clusters, Alifano et al. reported that 84%, and Smitt et al. reported that 73% were in clusters.^12^,^18^,^25^ The mean number of pneumothorax episodes per cluster were between 2.1±0.6 and 3.2±1.8.^12^,^13^,^22^,^23^,^25^ (Table-V) and in our study it was found to be 2.60±0.99 (88%), which is similar to the literature.

Different studies report different months and season frequencies. (Table-V) In our study, it was observed most in winter and spring, particularly in April. The reason is that, as can be understood from the results we obtained, during that period of the year, the temperature values considered critical (24.2°C and below for maximum temperature, 15.6°C and below for minimum temperature) are the figures that can be encountered more frequently.

A different view was put forward by Zhang et al.^14^ Since it was controversial which of the atmospheric parameters was, they tested the use of a local calendar. They related seasonal and lunar differences to important events in a local calendar, such as End of Heat (high), Great Snow (low), and Spring equinox (high).^14^ However, only three parameters were shown to be effective out of 24 sections, in this local calendar, raising questions about their overall effectiveness.

It has been accepted since ancient times that the moon affects many health events. It is also thought that the lunar cycle may be related to some diseases by affecting physiological systems.^2^ It is thought that this effect of the moon is associated with periodic changes in gravitational force and tidal events, major meteorological, biological, and other changes.^2^,^26^ It is sometimes thought to be related to mystical states. In a European country, it was determined that the moon affects health events by 10% in society and over 40% among health workers, surprisingly.^27^ In the literature, many studies have investigated the effects of the moon on cardiovascular, urological, psychiatric, gynecological diseases, surgical results, the number of emergency room visits, and lung diseases such as COPD.^1^,^2^,^5^,^6^,^8^,^27^ Most studies on the lunar cycle are not related to pneumothorax. Araz and Sok investigated the relationship between lunar days and spontaneous pneumothorax.^2^,^11^

Araz et al.^11^ examined a five-year period in their study including 131 patients but no significant result was obtained in terms of the relationship between the lunar cycle and pneumothorax. Sok et al.^2^ reported in their study of 244 cases covering 19 years that the cases were significantly more concentrated one week before and after the new moon, and that pneumothorax was detected most frequently on the 22nd lunar day and at least on the 17th lunar day. Pneumothorax was caused by obstruction following fluid imbalance in the small airways. In our study, pneumothorax cases were most frequently observed on the 28th lunar day and least frequently on the 13th lunar day, and an increase in the frequency of pneumothorax was observed between the 9th-11th, 20th-22nd, and 27th-29th lunar days. In terms of number and proportion of observations, it was observed most in the waning crescent stage and least in the first quarter stage, but no significant difference was found between the stages.

Araz et al.^11^ and Sok et al.^2^ used only the lunar day in their studies and did not analyze the effect of the phases, whereas in our study, the lunar cycle was divided into eight phases. Numerical dominance was observed in the lunar phases after the full moon and especially before the new moon, but it was not statistically significant. It is possible that changes in climate parameters during the lunar phases may affect the formation of spontaneous pneumothorax. However, there was no significant association between the lunar phases and the incidence of spontaneous pneumothorax in our study. We attribute this situation to the fact that the lunar effect did not create a strong enough difference in climate parameters to cause pneumothorax in our region, so much so that although there were numerical differences in pressure, temperature, wind, precipitation, and humidity between the lunar universes, there was no statistically significant difference. In addition, in our study, no relationship was found among solar and lunar events and the formation of pneumothorax.

We rarely come across studies that reveal a contradiction between superstitions and medical literature. One of these studies examined the zodiac signs and Friday the 13th; reported that superstitions had no effect on blood loss, frequency of emergency visits, gastrointestinal perforation, and aortic aneurysm rupture.^27^ Similarly, in our study, no relationship was found among zodiac signs, Friday the 13th, and the occurrence of spontaneous pneumothorax.

Only two studies have investigated the effect of the lunar cycle on pneumothorax. Compared to these studies,^28^-^30^ our study used more parameters, included a larger number of patients, a much longer time period, and employed multi-analysis. Furthermore, no other study has investigated the effect of astrological events and superstitions on pneumothorax. Our study included more parameters, a larger number of patients, and a longer time period than similar studies investigating the effect of climate parameters. Therefore, our study will be a reference for future researchers to compare with.

### Limitations:

It was a single-center study with a retrospective design. However, there was also an advantage to being a single center, since it was the reference clinic of the region studied, most initial applications and referrals came to our center, and most cases in the study region were in our clinic. We also provided standardization regarding the same climate conditions, industrial and air pollution conditions.

## CONCLUSION

As a result, the etiology of pneumothorax is multifactorial, so it would be wrong to hold only one parameter responsible. Atmospheric parameters have an effect, and therefore, their effects should be evaluated holistically. The effect of the moon on spontaneous pneumothorax appears to increase during certain periods, but more studies are needed to clarify the issue. Astrological conditions, zodiac date ranges, and superstitions are not associated with the occurrence of spontaneous pneumothorax.

### Authors’ Contribution:

**HFS:** Conceived, designed and did statistical analysis & editing of manuscript, did review and final approval of manuscript is responsible for integrity of research.

**HFS, AE & ST:** Did data collection and manuscript writing.
